# Comparative Analysis of Phenolics, Flavonoids, and Antioxidant and Antibacterial Potential of Methanolic, Hexanic and Aqueous Extracts from *Adiantum caudatum* Leaves

**DOI:** 10.3390/antiox4020394

**Published:** 2015-06-04

**Authors:** Dildar Ahmed, Muhammad Mehboob Khan, Ramsha Saeed

**Affiliations:** Department of Chemistry, Forman Christian College, Lahore-54600, Pakistan; E-Mails: mehboobkhankhokhar@gmail.com (M.M.K.); Ramsha.sd@gmail.com (R.S.)

**Keywords:** *Adiantum caudatum*, phenolics, flavonoids, antioxidant, antibacterial, Soxhlet extraction

## Abstract

In the quest for new medicines, the methanolic, hexanic, and aqueous extracts of *Adiantum caudatum* leaves, obtained by Soxhlet extraction, were analyzed for phenolic and flavonoid contents, and antioxidant and antimicrobial potential. TPCs (total phenolic content) of the methanolic, aqueous and hexanic extracts were 27.7, 21.1, and 16.7 μg of gallic acid equivalents per mL, respectively, while TFCs (total flavonoid content) were 13.2, 11.6, and 10.0 μg of rutin equivalents per mL, respectively. Antioxidant activities of the extracts in reducing power, FRAP (ferric reducing antioxidant power), phosphomolybdate and ABTS assays follow the same order of methanolic ˃ aqueous ˃ hexanic. In the DPPH assay, however, the aqueous extract exhibited a slightly higher antioxidant activity than the methanolic one. Methanol is therefore a better solvent to extract most of the antioxidant components from *A. caudatum* leaves. In lipid peroxidation inhibitory assay, the extracts showed almost similar behavior and their activity decreased gradually with time. The aqueous extract was the strongest inhibitor after two days, but the hexanic became the most potent after about three days. The antibacterial potential of the extracts was determined against *Bacillus subtilis*, *Escherichia coli* and *Pseudomonas aeruginosa*. Towards all the microbes, the aqueous extract was the most potent and the hexanic the least. *P. aeruginosa* was the most susceptible strain, while the aqueous and methanolic extracts exhibited a slightly higher efficacy against this pathogen than the drug amoxicillin. In conclusion, *A. caudatum* can potentially provide a remedy against disorders caused by oxidative stress and infections.

## 1. Introduction

*Adiantum caudatum* is an evergreen fern commonly called trailing maidenhair (family Adiantaceae). Adiantum, a large genus of about 200 species, is distributed globally from temperate to tropical regions and has many medicinal properties [1,2,3,4,5]. The fern *A. caudatum* (Syn. *A*. *incisum* Forssk) has been reported from northern hilly areas of Pakistan including Kashmir, Murree, Galliyat, Rawalpindi, and Mangora [6,7,8,9,10]. In folkloric medicine, it is used as a remedy to cure cough, diabetes, jaundice, fever, diarrhea, skin diseases, wounds, and as a natural antibiotic [2,4,5,10,11,12,13]. The plant has been shown to possess terpenoids and flavonoids [6,10,14].

Drug discovery is an ongoing requirement in order to find safe, effective, and affordable cures for an expanding spectrum of human ailments. Plants constitute a rich source of a wide variety of therapeutic molecules and therefore hold a great promise for new medicines. Natural antioxidants are required to prevent and/or cure the disorders caused by free radicals. The free radicals are highly reactive chemical species produced in the body and have the potential to damage cells, organelles, DNA, and other biomolecules, resulting in diseases such as cancer, and cardiovascular and neurodegenerative ailments [15]. The treatment of such diseases has serious efficacy and safety issues. In addition, it is often highly expensive and many people cannot afford it. This necessitates efforts to discover safe and effective remedies, readily available to common people. Treatment of infectious diseases is also becoming a challenge due to the problem of multi-drug resistance. As pathogens soon develop resistance to existing antibiotics, new alternatives are inevitable to treat infectious diseases [16]. It is therefore highly desirable to explore plants for new antimicrobial agents.

The present study was planned to investigate and compare antioxidant and antimicrobial activities of methanolic, aqueous and hexanic extracts from *Adiantum caudatum* leaves (fronds) using a hot extraction method employing a Soxhlet apparatus. As far as we could ascertain this is the first study of its kind on this plant.

## 2. Materials and Methods

### 2.1. Chemicals

The chemicals used in the present work were purchased from various companies. Sodium nitrite, potassium persulfate, dipotassium hydrogenphosphate, ferric chloride, monosodium dihydrogenphosphate, potassium ferricyanide, trichloroacetic acid, disodium hydrogenphosphate, Rutin, Mueller-Hinton agar (MHA), and Folin-Ciocalteu reagent were purchased from Merck (Darmstadt, Germany), gallic acid from Riedel-de-Haen (Seelze, Germany), ammonium molybdate, linoleic acid, ferrous chloride tetrahydrate and aluminum chloride from BDH Labs (Cambridge, England), DPPH (2,2-diphenyl-1-picrylhydrazyl) and ascorbic acid from MP Biomedicals (Illkirch, France), sodium acetate from Daejung (Siheung City, Korea), ammonium thiocyanate from Alfa-Aesar (Karlsruhe, Germany), amoxicillin from GlaxoSmithKline (Karachi, Pakistan) and trichloroacetic acid, TPTZ (2,4,6 tripyridyl-*s*-triazine), ABTS (2,2′-azino-bis(3-ethylbenzothiazoline-6-sulfonic acid) were purchased from Sigma-Aldrich (Steinheim, Germany).

### 2.2. Test Microorganisms

Test microorganisms were provided as a courtesy from Pharmagen Ltd. (Lahore, Pakistan) and included the following standard strains: *Bacillus subtilis* ATCC6633, *Escherichia coli* ATCC8739, and *Pseudomonas aeruginosa* ATCC9027.

### 2.3. Collection and Preparation of the Plant Material

The aerial parts of the fern *Adiantum caudatum* were collected from the hilly area near Abbottabad, Pakistan. The leaves were carefully separated, washed with distilled water, and then dried under shade for two weeks. The dried leaves were crushed and ground with a coffee blender to obtain a powder. A Soxhlet apparatus was used for extraction into three solvents, methanol, hexane, and water. To obtain methanolic extract, 30 g of the powdered plant material and 25 mL of the solvent were loaded in the apparatus and refluxed for 6 h on a hotplate. To ensure maximum extraction, the process was repeated twice. Hexanic and aqueous extracts were obtained in the same manner. The solvents were then evaporated under reduced pressure using a rotary evaporator to obtain extracts as semi-solid materials [17,18].

### 2.4. Antioxidant Properties

#### 2.4.1. Total Phenolic Content

Total phenolic content (TPC) of each of methanolic, hexanic, and aqueous extract obtained by hot extraction with a Soxhlet apparatus was estimated according to the method of Slinkard *et al.* [19]. Each plant sample was prepared by dissolving 4.3 mg in 10 mL methanol. The mixture was sonicated for 5 min to obtain a homogenized solution. To 300 μL of this solution taken in a test tube, 1 mL methanol, 3.16 mL distilled water and 200 μL Folin-Ciocalteu reagent were added. Then, after an 8 min incubation at room temperature, 600 μL sodium carbonate solution (10%) was added and the test tube was covered with aluminum foil and incubated in a hot water bath at 40 °C for 30 min. A blank was prepared using the same procedure but replacing the plant extract with an equal volume of methanol. The absorbance of the sample was determined using a UV visible spectrophotometer at 765 nm. The standard curve of gallic acid was obtained using the same procedure. Total phenolic content was expressed as μg of gallic acid equivalents (GAE) per mL, which was calculated using the formula, *y* = 0.0425 *x* − 0.0247, where, *y* is the absorbance at 765 nm and *x* is the amount of gallic acid equivalent (μg/mL).

#### 2.4.2. Total Flavonoid Content

The total flavonoid content (TFC) was determined using a reported protocol [20]. A solution of each extract was prepared by sonicating 3 mg in 10 mL methanol for 10 min. To 300 μL extract (0.3 mg/mL in methanol) in a test tube, 3.4 mL aqueous methanol (30%) was added to obtain a clear solution. Then, 150 μL aqueous sodium nitrite solution (0.5 M) was added followed by 150 μL aluminum chloride solution (0.3 M). After 5 min, 1 mL sodium hydroxide solution (1 M) was added, and the content was mixed well before measuring its absorbance at 506 nm on a UV visible spectrophotometer against a blank, which was prepared by the same procedure except replacing the plant extract with an equal volume of methanol. Similarly, a calibration curve of rutin was obtained (for concentrations ranging from 75 mg/L to 750 mg/L) and the total flavonoid content of each extract was expressed as μg of rutin equivalents (RE) per mL, calculated using the formula, *y* = 0.0236 *x* + 0.0348, where, *y* is the absorbance at 506 nm and *x* is the amount of rutin equivalent (μg/mL).

#### 2.4.3. Reducing Power Assay

Antioxidant capacity as per reducing power assay was measured according to a method reported by Oyaizu [21]. Briefly, a set of 5 dilutions of each plant extract was prepared in 50% aqueous methanol ranging from 5 mg/L to 25 mg/L. In a test tube, 2.5 mL plant extract, 2.5 mL sodium phosphate buffer (0.2 M, pH 6.6) and 2.5 mL potassium ferricyanide (1% w/v in distilled water) were added and mixed well. The mixture was incubated in a water bath for 20 min at 50 °C. Then, 2.5 mL trichloroacetic acid (10% w/v in distilled water) was added, and the mixture was centrifuged at 650 rpm for 10 min. The supernatant (5 mL) was taken into a test tube and 5 mL distilled water and 1 mL ferric chloride (0.1% w/v in distilled water) solution was added and mixed well. Absorbance was measured at 700 nm. Blank for each solvent was run using the same procedure but replacing the plant extract with an equal volume of solvent.

#### 2.4.4. Ferric Reducing Antioxidant Potential (FRAP)

Ferric reducing antioxidant potential (FRAP) of the extracts of *A. caudatum* was measured according to the method proposed by Benzie and Strain [22]. FRAP reagent was prepared by mixing in 25 mL acetate buffer (30 mM; pH 3.6), 2.5 mL TPTZ solution (10 mM) and 2.5 mL ferric chloride solution (20 mM). The mixture was incubated for 15 min at 37 °C before use. Ascorbic acid (vitamin C) was employed as a standard in this assay, and its calibration curve was obtained by using its concentrations ranging from 50 mg/L to 500 mg/L in water. To 2.85 mL FRAP reagent in a test tube, 150 μL plant sample (0.1 mg/mL, in methanol) or standard was added. The mixture was incubated for 30 min in the dark, and its absorbance was measured at 593 nm. The blank contained an equal volume of methanol instead of the plant sample. The results were reported as μg of ascorbic acid equivalents (AAE) per mL.

#### 2.4.5. Phosphomolybdate Assay for Total Antioxidant Capacity

To determine total antioxidant capacity (TAC) of *A. caudatum* extracts as per phosphomolybdate assay proposed by [23], the procedure described by [24,25] was used with slight modification. For sample preparation, 250 μg plant extract was dissolved in 1 mL methanol and sonicated for 5 min to get a homogeneous mixture. Ascorbic acid was used as a standard. A stock solution of ascorbic acid (5000 mg/L) was prepared in distilled water, from which dilutions were made ranging from 25 mg/L to 500 mg/L.

In a test tube, 300 μL plant extract was mixed with 3 mL phosphomolybdate reagent (0.6 M sulfuric acid, 28 mM sodium phosphate and 4 mM ammonium molybdate). The test tube was covered with aluminum foil and incubated at 95 °C for 90 min. The mixture was then allowed to reach room temperature when its absorbance was recorded at 765 nm. Blank was run using the same procedure but containing an equal volume of methanol in place of the plant sample. The antioxidant capacity was reported as μg of ascorbic acid equivalents (AAE) per mL.

#### 2.4.6. DPPH Radical Scavenging Activity

The DPPH free radical scavenging activity of methanolic, hexanic, and aqueous extracts of *A. caudatum* was determined according to the method reported by Brand-Williams *et al.* [26] with slight modification [27]. The stock solution of the radical, prepared by dissolving 24 mg DPPH in 100 mL methanol, was kept in a refrigerator until further use. The working solution of the radical was prepared by diluting the DPPH stock solution with methanol to obtain an absorbance of about 0.98 (±0.02) at 517 nm [28].

In a test tube, 3 mL DPPH working solution was mixed with 100 μL plant extract (1 mg/mL) or the standard solution. The absorbance was measured at 517 nm for a period of 30 min. The percent antioxidant or radical scavenging activity was calculated using the following formula:

%Antioxidant activity = [(Ac − As)/Ac] × 100

where, Ac and As are the absorbance of control and sample, respectively. The control contained 100 μL methanol in place of the plant sample.

#### 2.4.7. ABTS^•+^ Decolorization Assay

Antioxidant activity of *A. caudatum* extracts as per ABTS^•+^ decolorization assay was measured using the method reported by Re *et al.* [29] with some modifications [30]. The working solution of ABTS^•+^ radical was made by reacting ABTS (9.5 mL, 7 mM) with potassium persulfate (245 μL, 100 mM), and raising the volume to 10 mL with distilled water. The solution was kept in the dark at room temperature for 18 h, and then diluted with potassium phosphate buffer (0.1 M, pH 7.4) to an absorbance of 0.70 (±0.02) at 734 nm. Plant samples were prepared in methanol with dilutions 50–1250 μg/mL. A sample (10 μL) was placed in a test tube and mixed thoroughly with 2.99 mL ABTS radical working solution. Absorbance of the resulting clear mixture was recorded at 734 nm. The percent antioxidant activity of the sample was determined using the following formula:

%Antioxidant activity = [(Ac − As)/Ac] × 100

where Ac and As are the absorbances of the control and sample, respectively. The control was prepared by adding 10 μL of methanol in place of the sample.

#### 2.4.8. Lipid Peroxidation Inhibitory Assay

The lipid peroxidation inhibitory activity of the extracts of *A. caudatum* was determined using the method described by Mitsuda *et al.* [31]. Linoleic acid (a doubly unsaturated fatty acid) emulsion was used as a substrate, which was prepared by mixing 155 μL linoleic acid and 175 μg Tween-20 in 50 mL phosphate buffer (pH 7; prepared by dissolving dipotassium hydrogenphosphate in distilled water) followed by sonication. The plant sample was prepared by mixing 10 mg plant extract in 2 mL methanol followed by sonication for about 5 min to obtain a homogenized solution. To 100 μL plant extract (5 mg/mL), 2.4 mL phosphate buffer and 2.5 mL linoleic acid emulsion were mixed. The mixture was then incubated for 25 min at 37 °C. A sample (100 μL) of this solution was taken after every 24 h and mixed with 3.7 mL ethanol and 100 μL ferrous chloride solution (20 mM; prepared by dissolving 0.03976 g ferrous chloride in 10 mL 3.5% HCl). The contents were mixed well before treating with 100 μL potassium thiocyanate solution (30% in distilled water). The absorbance of the resulting clear solution was recorded at 500 nm. A solution containing 2.5 mL linoleic acid emulsion and 2.5 mL phosphate buffer was run as a blank.

### 2.5. Antimicrobial Activity

The antibacterial activity of methanolic, hexanic, and aqueous extracts of *A. caudatum* was determined by the common agar well diffusion method [32] with slight modification. Each of the plant samples was prepared by dissolving 40 mg extract in 1 mL DMSO. Amoxicillin was used as the standard drug. The autoclave (at 121 °C) periods for media preparation and Petri plates were 15 min and 30 min, respectively. McFarland solution (0.5%) was prepared by mixing 99.5 mL sulfuric acid (0.18 M) and 0.5 mL barium chloride solution (0.18 M), and adjusting the absorbance at 0.08 to 0.1 at 600 nm. The solution was kept in the dark and sealed. Media agar was prepared by dissolving 3.2 g Mueller-Hinton agar in 100 mL distilled water followed by autoclaving for 15 min at 121 °C. Inoculum was prepared by adding 3 mL normal saline (0.9%) in slants containing bacterial cultures and was mixed in circular motion so that all the bacterial cultures were transferred into the normal saline. The turbidity was adjusted by adding saline water according to 0.5% McFarland standard. In a flask, 100 mL prepared media agar was taken, and used as seed agar for bacterial cultures. The temperature of seed agar was maintained at 50 °C to ensure that it would evenly spread on media plates. The inoculum was poured into the seed agar and was shaken well for even mixing. In 100-mm autoclaved Petri plates, 21 mL prepared media agar was poured with the help of a pipette. Immediately after the plates became solidified, 4 mL seed agar was added. In this way, 25 mL standard volume of medium in the Petri plates was attained. In order for the agar to set, the plates were refrigerated for 1 h. Then, holes were made in the plates (using an 8-mm puncture) and were labeled. Samples were added in to the holes, and kept incubated for 24 h at 35 °C. At the end, zones of inhibition were carefully measured.

### 2.6. Statistical Analysis

All the determinations were conducted at least three times (*n* = 3); the statistical mean was calculated with ± SD using Excel 2013 (Microsoft Corporation, Redmond, WA, USA).

## 3. Results and Discussion

The finely divided powder of the shade-dried leaves (15 days) of the fern *A. caudatum* was subjected to hot extraction using a Soxhlet apparatus. As a result, methanolic, hexanic, and aqueous extracts were obtained. The extracts were analyzed for antioxidant and antibacterial activities. A number of assays were conducted to analyze antioxidant activities of the extracts since antioxidant activities of different types of substances involve different mechanisms, and no single assay is conclusively applicable to all of them.

### 3.1. Antioxidant Properties

#### 3.1.1. Total Phenolic Content and Total Flavonoid Content

**Figure 1:**
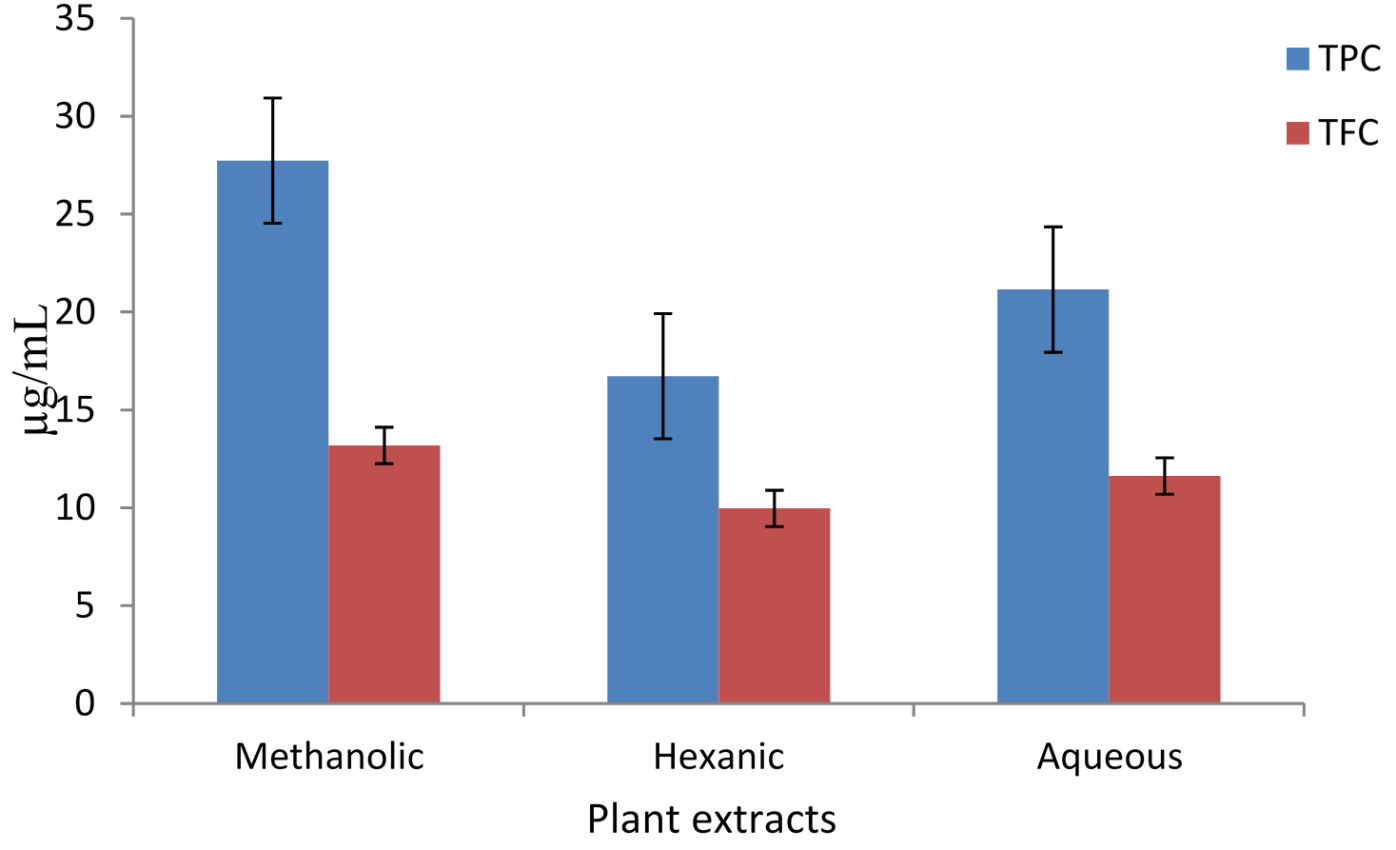
Total phenolic content (TPC) and total flavonoid content (TFC) of different extracts of *Adiantum caudatum* expressed as μg GAE/mL and μg RE/mL, respectively (*n* = 3).

The total phenolic content (TPC) of methanolic, hexanic, and aqueous extracts from *A. caudatum* were determined in terms of μg of Gallic Acid Equivalents per mL (μg GAE/mL), and the results are displayed in Figure 1. The methanolic extract had the highest TPC followed by the aqueous extract, with the hexanic extract showing the lowest TPC. The total flavonoid content (TFC) of the different extracts of *A. caudatum* were determined in terms of μg of Rutin Equivalents per mL (μg RE/mL), and the results are shown in Figure 1. Methanolic extract had the highest TFC followed by aqueous extract.

Under the basic reaction conditions, a phenol loses an H^+^ ion to produce a phenolate ion, which reduces Folic-Ciocalteu reagent [33,34]. The change is monitored spectrophotometrically. As phenolics (including many flavonoids) contain polar phenolic hydroxyl group/s, their high extraction into methanol and water is quite reasonable. Likewise, the lowest TPC, of the hexanic extract may also be explained on the same grounds. Many flavonoids have aprotic, ether (e.g., methoxy) group/s rather than protic, hydroxyls. As a result, they may also appear in the hexanic extract. The basis of the total flavonoid assay is the fact that aluminum ion (Al^3+^) forms complexes with C-4 keto and either C-3 or C-5 hydroxyl, or with ortho hydroxyl groups in the A or B ring [35]. In each solvent, the TPC is higher than the TFC, supporting the fact that most flavonoids are also phenolics. In conclusion, methanol was a better solvent for extraction of both phenolics and flavonoids.

#### 3.1.2. Reducing Power

**Figure 2:**
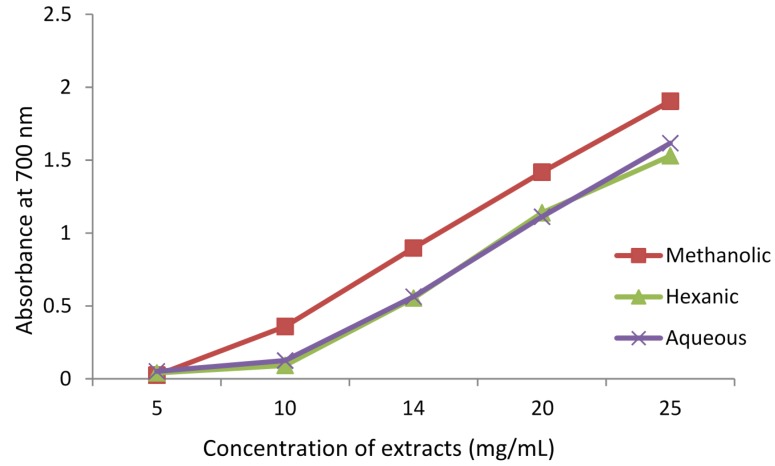
Reducing power assay of different extracts of *Adiantum caudatum* expressed as absorbance at 700 nm (*n* = 3).

Reducing power (or, antioxidant capacity) of the methanolic, hexanic, and aqueous extracts of *A. caudatum*, was determined and the results are shown in Figure 2.The methanolic extract displayed the highest reducing power, while the hexanic and aqueous extracts showed almost equal potency. All three extracts showed almost similar increasing trend in reducing power with the increase in extract concentration. In this assay, the presence of reducers (*i.e.*, antioxidants) causes the reduction of the Fe^3+^/ferricyanide complex to the ferrous form. Therefore, measuring the formation of Perl’s Prussian blue at 700 nm can monitor the Fe^2+^ concentration [36]. The methanolic extract which had the highest TPC and TFC, also displayed the highest reducing power (Figure 1). The correspondence alluded to the fact that phenolics and flavonoids possess high antioxidant potential.

#### 3.1.3. Ferric Reducing Antioxidant Potential (FRAP)

**Figure 3:**
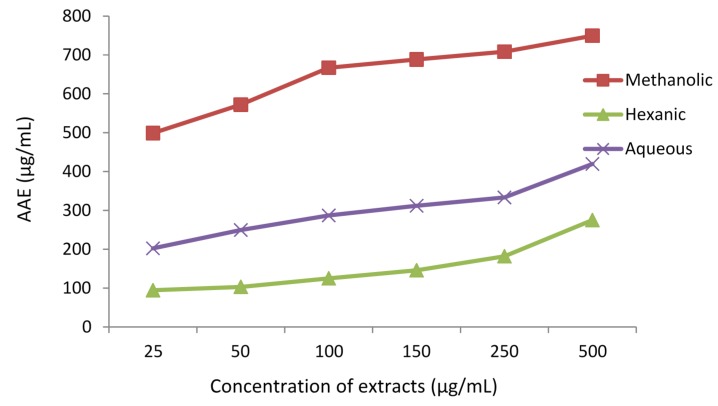
Ferric reducing antioxidant potential (FRAP) values of different extracts of *Adiantum caudatum* in terms of ascorbic acid equivalents (AAE) (*n* = 3).

Antioxidant capacity of extracts was determined using FRAP assay (Figure 3). In this assay, ferric ions are reduced to ferrous ions in the presence of an antioxidant (or, a reducing agent) which form a blue-colored ferrous tripyridyltriazine complex (Fe^2+^-TPTZ) at pH 3.6. The change is monitored spectrophotometrically at 593 nm [33]. The methanolic extract displayed the highest antioxidant capacity, followed by the aqueous and hexanic extracts. All the three extracts showed similar increasing trend in activity with increase in extract concentration. There was a strong correlation between TPC, TFC and FRAP values (Figure 1 and Figure 3) supporting the fact that phenolics and flavonoids are highly potent antioxidants.

#### 3.1.4. Total Antioxidant Capacity as per Phosphomolybdate Assay

**Figure 4:**
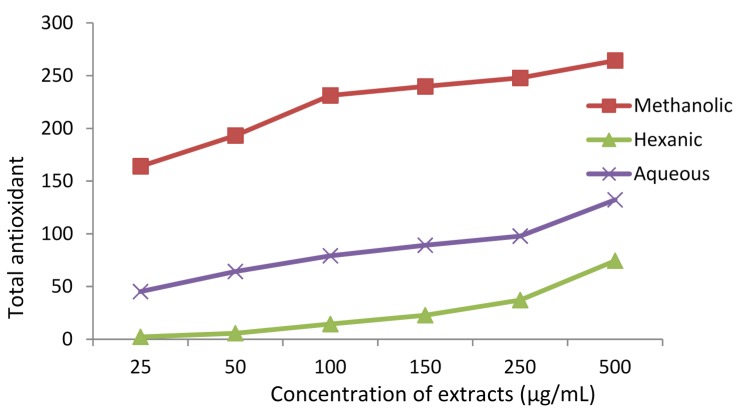
Total antioxidant capacity of different extracts of *Adiantum caudatum*, according to phosphomolybdate assay, expressed as μg/mL of ascorbic acid equivalents (AAE), (*n* = 3).

Total antioxidant capacity (TAC) of the methanolic, hexanic and aqueous extracts was determined using phosphomolybdate assay (Figure 4). The assay is based on the fact that molybdenum(VI) is reduced to molybdenum(V) in the presence of a reducing agent (antioxidant), forming a green phosphomolybdate(V) complex, which can be evaluated spectrophotometrically at 765 nm [24,25]. The assay involves an electron transfer (ET) mechanism. Many natural products, including phenols and flavonoids, can cause this reduction. As Figure 4 reveals, the antioxidant activity is dose-dependent. The methanolic extract showed a much higher activity than the other two extracts, while the hexanic extract was the least active. This order of activity from polar methanolic to nonpolar hexanic extract is quite normal since the polar solvents have a much stronger ability to dissolve and hence extract polar phytochemicals. TPC and TFC followed the same order, which supported our conclusion.

#### 3.1.5. DPPH Radical Scavenging Activity

**Figure 5:**
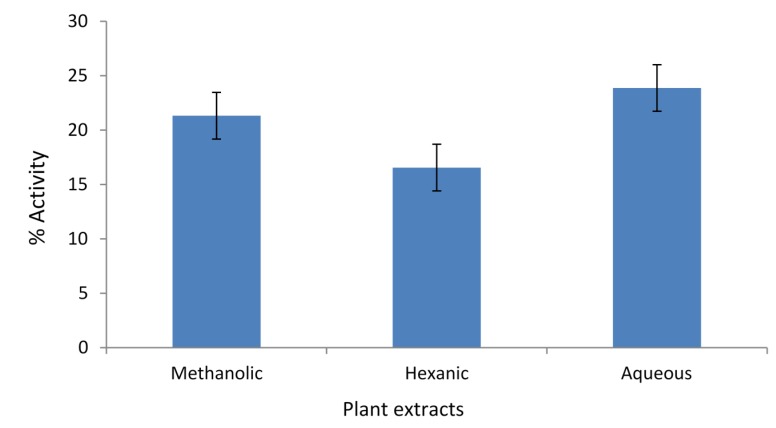
Percent free radical scavenging activities of methanolic, hexanic and aqueous extracts (concentration 1 mg/mL) of *Adiantum caudatum* as per DPPH assay after an incubation of 30 min (*n* = 3).

**Figure 6:**
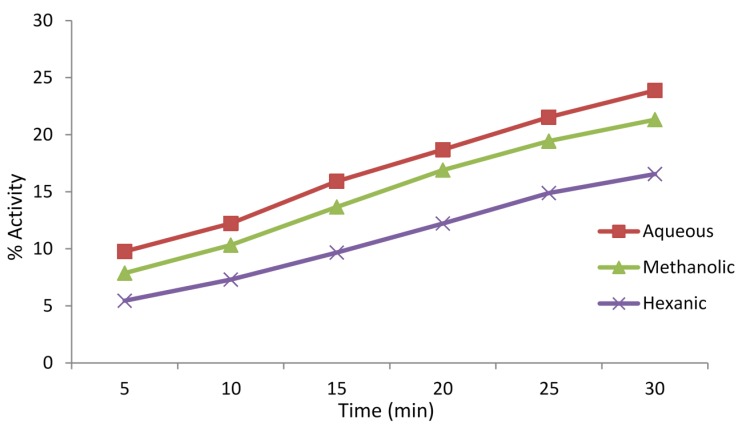
Percentage radical scavenging activities of methanolic, hexanic and aqueous extracts (concentration 1 mg/mL) of *Adiantum caudatum* as per DPPH assay as a function of time for 30 min (*n* = 3).

Antioxidant, or free radical scavenging, activities of the extracts of *A. caudatum* were determined using DPPH radical scavenging assay. The results are displayed in Figure 5. In the assay, the aqueous extract showed a slightly higher activity than the methanolic extract, which, in turn, was more active than the hexanic extract. This means phytochemicals soluble in water possess a stronger potential to scavenge DPPH free radicals. The antioxidant activity was also determined as a function of time and found to increase gradually with time (Figure 6). The DPPH radical is capable of accepting an electron as well as a hydrogen but data supports the latter mechanism as a predominant, if not exclusive, pathway [37,38,39]. The slightly higher free radical scavenging activity of the aqueous extract presumably indicate the presence of a higher content of protic flavonoids in the aqueous extract than the methanolic and hexane extracts, facilitating HAT (hydrogen atom transfer) to take place. Steric inaccessibility of the large molecules may also be a factor [40].

#### 3.1.6. Antioxidant Activity as per ABTS^•+^ Radical Decolorization Assay

**Figure 7:**
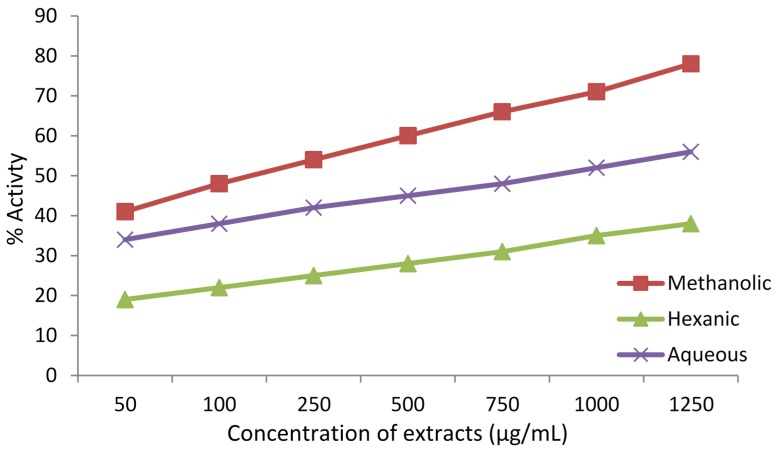
Antioxidant capacity of the extracts of *Adiantum caudatum* as per ABTS^•+^ radical assay expressed as percentage activity (*n* = 3) as a function of concentration of extracts.

Antioxidant activity was expressed as percentage [38,41]. The results are shown in Figure 7. The methanolic extract displayed the highest antioxidant capacity in this assay, followed by the aqueous and hexanic extracts. All three extracts showed a similar increasing trend in antioxidant activity with an increase in their concentrations.

#### 3.1.7. Lipid Peroxidation Inhibitory Activity

**Figure 8:**
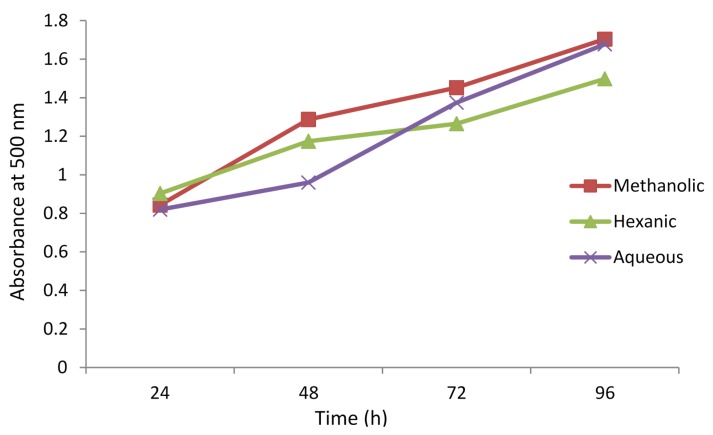
Lipid peroxidation inhibitory activity of different extracts of *Adiantum caudatum* as a function of time (*n* = 3).

The ability of the extracts of *A. caudatum* to inhibit peroxidation of lipids was evaluated using lipid peroxidation inhibitory assay. The results are displayed in Figure 8. In the assay, linoleic acid undergoes auto-oxidation producing radicals. These radicals are scavenged (reduced) by the reagent ferrous ions, which are oxidized to ferric ions. The ferric ions, then, form a colored complex with SCN^−^ ions, which is monitored spectrophotometrically at 500 nm [31]. The presence of an antioxidant in the medium inhibits auto-oxidation of linoleic acid, resulting ultimately in low production of the ferric thiocyanate complex. Low absorbance, therefore, means a high peroxidation inhibitory activity of a sample. The extracts showed almost similar behavior; their activity decreased gradually with time and at the end of 96th hour it was much lower than at the beginning. The aqueous extract was the strongest inhibitor after two days, but hexane became the most potent after about three days.

### 3.2. Antibacterial Activity

**Figure 9:**
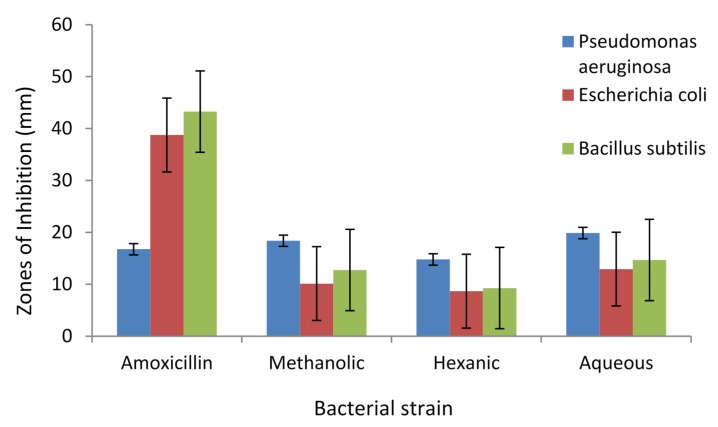
Zones of inhibition (mm) of different extracts of *Adiantum caudatum* at 40 mg/mL concentration against three bacterial strains in comparison with Amoxicillin (*n* = 3).

Antibacterial activities of methanolic, hexanic and aqueous extracts of *A. caudatum* were determined against three standard bacteria in terms of zones of inhibition (mm). The common antibiotic Amoxicillin was used as a positive control. The results are displayed in Figure 9.

*Pseudomonas aeruginosa* was the most susceptible, and the aqueous and methanolic extracts exhibited a slightly higher efficiency against this pathogen than the drug amoxicillin. As Figure 9 reveals, not only was the antibiotic less effective against this microbe, but the extracts also had higher efficacy against it. The order of potency of the extract remained the same towards all the microbes; the aqueous being the most potent and the hexanic the least. An important inference that may be drawn is that, subject to further investigation and clinical trial, an aqueous decoction of the plant may have the potential to cure infections caused by *P. aeruginosa*. Perhaps this provides an explanation for the use of the plant for the treatment of skin diseases in ethnomedicine [2,4].

## 4. Conclusions

The study showed that *Adiantum caudatum* possesses considerable antioxidant activities. The most antioxidant compounds are extracted in the methanol, and the methanolic extract showed the highest activity in most of the assays. There was a strong correlation between phenolics and antioxidant activity substantiating the well-known free radical scavenging potential of these natural products. The plant extract showed a remarkable inhibiting activity against the pathogen *Pseudomonas aeruginosa.* This work will provide a foundation for further phytochemical and pharmacological studies.
